# Peculiar Effect of Corrosive Sublimate

**Published:** 1886-02

**Authors:** 


### A Peculiar Effect of Corrosive Sublimate.

The bichloride of mercury is at present used a great deal for disinfecting purposes, and it is especially employed in large hospitals, where strict antisepsis is faithfully carried out. It has always been maintained that the drug, if diluted in the usual manner, caused no bad after-effects. Generally 1 in 1,000 is the solution applied to wounds, while 1 in 4,000 answers all antiseptic purposes. But that the first solution is not so innocuous as most seem to believe, is plainly proven by the following case:

Dr. J. C. Biddle, the well-known surgeon-in-chief of the State Hospital at Ashland, who is perhaps to-day, outside of Philadelphia, the most reliable authority in our State on injuries, treats all his numerous surgical cases on strictly aseptic principles. Last week he had a patient with a lacerated wound, extending from the upper part of the thigh to below the knee, and connected with two large pus cavities. After these had been laid open, they were thoroughly washed out twice daily with a corrosive sublimate solution of 1 in 1,000. By the twelfth day, the thermometer showed an increase of 4° above normal, which temperature soon became continuous. The patient besides suffered from a severe diarrhœa, which did not yield to any treatment. Finally Dr. Biddle, on reflection, concluded that the symptoms, though greatly resembling septicæmia, were really caused by corrosive sublimate poisoning. He at once ordered the substitution of carbolic acid for the bichloride, with the result that within twenty-four hours the diarrhœa ceased and the temperature became about normal. About the same time, Dr. Biddle made a similar observation in a milder case, so that there is no doubt of the causal relation between the drug and the morbid symptoms mentioned. As a contribution to the literature on the subject the facts quoted are decidedly of great interest.

